# 2596. Title: The Dynamics Of Invasive Pneumococcal Disease (IPD) due to *Streptococcus pneumoniae* and Impact Of Pneumococcal Conjugate Vaccines (PCVs) In Canada From 2000-2019

**DOI:** 10.1093/ofid/ofad500.2211

**Published:** 2023-11-27

**Authors:** Bernice N Ramos, Nirma Khatri Vadlamudi, Irene Martin, Greg Tyrrell, Nicholas Brousseau, Averil M Griffith, Manish Sadarangani

**Affiliations:** University of British Columbia, Vancouver, British Columbia, Canada; The University of British Columbia, Vancouver, British Columbia, Canada; National Microbiology Laboratory (NML), Winnipeg, MB, Canada; UAlberta, Edmonton, Alberta, Canada; Institut national de santé publique du Québec, Québec, Quebec, Canada; National Microbiology Laboratory, Winnipeg, Manitoba, Canada; University of British Columbia, Vancouver, British Columbia, Canada

## Abstract

**Background:**

IPD remains a major cause of morbidity and mortality globally despite the implementation of PCVs in childhood immunization programs, due to the emergence of non-vaccine serotypes (NVTs) and persisting vaccine types. Newly approved vaccines such as PCV15 and PCV20 may address some of these issues. This study aimed to describe the epidemiology of IPD in Canada from 2000-2019 after PCV7 and PCV13 approval in 2001 and 2010, respectively, and evaluate the potential future impact of PCV15 and PCV20.

**Methods:**

Data were collected from IPD cases in children and adults captured by the Pneumococcal Reference Laboratory in Alberta and the National Microbiology Lab during 2000-2019; this includes the majority of isolates from IPD across Canada, excluding Quebec. We calculated annual age-, and serotype-specific incidence rates and serotype distribution using population estimates from Statistics Canada.

**Results:**

A total of 37,921 isolates from IPD cases were included in the analyses. After PCV7 introduction, IPD due to PCV7 serotypes decreased by 64% (2.2 to 0.8 cases per 100,000 population per year) from 2001 to 2010, while PCV13/non-PCV7 serotypes increased between 2004 and 2010 from 1.0 to 3.8 per 100,000 per year. After PCV13 introduction, IPD due to PCV13/non-PCV7 serotypes decreased by 53% (3.8 to 1.8 per 100,000 per year) between 2010 and 2019. The overall rate initially decreased from 8.7 to 7.6 per 100,000 between 2010-2014 but increased to 9.6 per 100,000 in 2019. From 2010 to 2019, IPD incidence increased by 63% (0.8 to 1.3 per 100,000 per year) for PCV15/non-PCV13 serotypes and by 33% (1.2 to 1.6 per 100,000) for PCV20/non-PCV15 serotypes. The proportion of serotypes in 2019 covered by PCV15 and PCV20 was 32% and 45% in infants aged < 2 years and 43% and 56% in adults aged ≥65 years, respectively. There was an increased IPD burden observed for some PCV13 serotypes: serotype 3 increased from 0.7 to 1.1 per 100,000 per year between 2010 and 2019 and serotype 4 increased from 0.2 to 0.8 per 100,000 per year between 2014 and 2019.

**Conclusion:**

IPD rates in Canada continue to increase despite the widespread use of PCV13. While PCV15 and PCV20 hold promise in addressing prevalent recently circulating serotypes, there remains a need to develop vaccines with broader coverage considering the constant emergence of NVTs.

**Disclosures:**

**Nirma Khatri Vadlamudi, PhD, MPH**, Pfizer Canada: Grant/Research Support **Greg Tyrrell, PhD**, Merck Canada: Advisor/Consultant|Merck Canada: Grant/Research Support **Manish Sadarangani, BM BCh, FRCPC, DPhil**, GlaxoSmithKline: Grant/Research Support|Merck: Grant/Research Support|Moderna: Grant/Research Support|Pfizer: Grant/Research Support|Sanofi Pasteur: Grant/Research Support|Seqirus: Grant/Research Support|Symvivo: Grant/Research Support|VBI Vaccines: Grant/Research Support

